# Low voltage environmentally friendly plasma electrolytic oxidation process for titanium alloys

**DOI:** 10.1038/s41598-022-09693-w

**Published:** 2022-04-11

**Authors:** Fengyan Hou, Rukmini Gorthy, Ian Mardon, Da Tang, Chris Goode

**Affiliations:** 1 Cirrus Materials Science Ltd., 5B Target Ct., Auckland, 0627 New Zealand; 2 Department of Chemical and Materials Engineering, The University of Auckland, 20 Symonds Street, Auckland, 1010 New Zealand

**Keywords:** Corrosion, Photocatalysis, Composites

## Abstract

Plasma electrolytic oxidation (PEO) is a surface-treatment process extensively used to protect the surfaces of light metals such as Mg, Al, and Ti. Here, we report an environmentally friendly PEO process that uses nitrogen-containing electrolytes and low voltages (120 V) to form ~ 12 micron thick, uniform, adherent and porous oxide coatings on T1 titanium alloy surfaces. We evaluated the influence of nitrogenation by comparing the coatings to alloys treated in PEO baths without nitrogen-containing compounds. Both sets of samples exhibited basalt-like morphologies with distinct variation in the pore structures. The composition analyses showed that the coatings were primarily composites of titanium oxides and silicates. The T1 Ti alloys treated with nitrogen-containing electrolytes also contained TiC and TiN. This is the first ever report of producing Ti_x_O_y_, Ti–Si–O, TiC, and TiN composite coatings using a single PEO bath without carbide/nitride nanoparticles. The bandgaps of the coatings suggested visible light functionality. The use of nitrogen-based compounds in the PEO baths improved the hardness of the oxide layers but introduced stress-induced cracking which are potentially responsible for the reduction in corrosion resistance of the nitride and carbide containing coatings.

## Introduction

Plasma electrolytic oxidation (PEO) is a surface-treatment process used to protect metallic substrates by inducing the formation of environmentally inert oxide coatings. The process applies a high direct current (DC), pulsed DC or alternating current (AC) voltage between a stable cathode and the target substrate in an electrolytic bath. Micro-arc discharge channels on the substrate surface form the oxide coating. The primary objective of PEO surface treatments is to provide metallic substrates with wear and corrosion resistance. A unique feature of the PEO process is that plasma thermochemical interactions at multiple-surface discharges result in the bimodal growth of oxide coatings. The PEO treatment processes simultaneously form a porous layer on the substrate which extends a few microns into the bulk of the substrate forming a highly adherent corrosion resistant coating. Magnesium, aluminium, and titanium alloys are among light metals commonly subjected to PEO surface treatments^1^. Recent reviews also report the use of PEO surface treatments for copper, zinc and niobium alloys^2^.

Titanium and its alloys have a diverse range of applications in aerospace, chemical, biomedical^3,4^ and semiconductor industries because of their high specific strength, low density, ease of fabrication and biocompatibility. T1 titanium alloys are of particular interest for scientific^5^ and industrial applications as they possess lower elasticity and superior corrosion resistance compared to other titanium alloys. T1 alloys are not directly treatable with standard surface engineering processes as they neither respond to thermal hardening processes nor are they reactive with oxygen. Thermochemical surface treatment processes available for enhancing the wear and corrosion resistance of Ti alloys include physical vapor deposition (PVD), chemical vapor deposition (CVD), chemical conversion, anodization, electroplating, electroless plating, polymerization of organic coatings and PEO. Surface treating titanium alloys by PEO is a well-known, commercially viable process as PEO can develop thick, adherent, wear resistant oxidized coatings on the alloy surface. Other surface-treatment processes for titanium alloys are carburizing and nitriding. Carbonization of titanium alloys produces a micron-scale TiC coating and requires high processing temperatures (> 1000 °C)^6^. Nitriding titanium alloys results in the formation of extremely hard Ti–N coatings (1500 to 3000 HV), and the process requires controlled N_2_-rich environments^7^. The downside to direct nitridation of titanium alloy surfaces is the reduction of their fatigue strength^8^.

PEO coatings on titanium are of high interest due to their ductility, corrosion resistance, and especially photocatalytic nature. Scientific interest in titanium oxides and titanium oxide-based materials was piqued by the discovery of the ability of TiO_2_ to split water under UV-light^9^. This discovery prompted a vast amount of research on Ti_X_O_Y_-based photocatalysts for self-cleaning applications, energy harvesting applications and organic pollutant decomposition^10^. Most TiO_2_-based materials reported in the literature are nanoparticles and require additional processing for real world applications. Efficient functionalization of titanium oxides also requires the materials to be both responsive under visible light and mechanically robust.

Functionalization techniques for Ti–O-based materials include process-induced nanostructured arrays, surface crystallization, and micro/nanoparticle incorporation into PEO-treated ceramic coatings. Uhm et al., reported producing PEO-tailored titania nanotubes sputtered with Ag for antimicrobial applications. The authors reported that the functionalized coatings reduced *S. aureus* cell viability^11^. Jiang et al., produced nickel titanate nanowires with controllable sizes for catalytic CO oxidation^12^. Ti alloys undergo surface crystallization when treated with plasma techniques for surface protection. Benčina et al., report that controlling plasma conditions can induce phase-initiation or transition between anatase and rutile TiO_2_^3^. Studies show multi-phase TiO_2_ coatings exhibit enhanced photocatalytic and energy harvesting applications when compared to pure phase anatase or rutile-based coatings^13^. Addition of micro/nanoparticles to PEO baths is one of the more recent techniques for functionalizing surface treated T1 Ti alloys. Incorporating nanoparticles such as Ag, Cu and Zn^14^ into PEO-treated Ti alloys by electrolyte modification improved the antibacterial performance, corrosion resistance behaviour and bio-integration applicability of the materials. Non-metallic particles such as SiO_2_, Si_3_N_4_, SiC, Al_2_O_3_ and ZrO_2_ and graphene nanoparticles reportedly improved the mechanical functionality of Ti-alloyed components for aerospace applications. Literature studies also report incorporating NiO, Fe_2_O_3_, Cr_2_O_3_, and hydroxyapatite^15^ microparticles into the electrolytic baths to produce functional ceramic oxide coatings on titanium and/or titanium alloys.

Silicate compounds are commonly used constituents for PEO electrolytes, and few studies report the combined influence of these compounds on functionalizing ceramic oxide coatings formed by surface treating T1 alloys^16^. PEO-coated titanium implants, prepared using a silicate containing electrolyte, exhibited higher osseointegration in rat models than raw-titanium implants^17^. Shokoufar et al., reported enhanced mechanical behaviour of PEO-treated Ti–6Al–4V surfaces. The authors incorporated carbide content into the ceramic coating by adding SiC nanoparticles to the PEO electrolyte. The study also showed that incorporation of nanoparticles into the PEO-treated coatings negatively impacted the friction coefficient and corrosion current density of the oxidized materials^18^. Chen et al., reported a two-step process of producing carbon incorporated PEO treated titanium oxide coatings with improved tribological behaviour. The authors used magnetron sputtering to deposit diamond-like carbon into the micropores of PEO-treated commercial grade-2 titanium metal^19^. Nitrogenation of TiO_2_-based ceramic coatings is another well-researched method for improving their functionality; however, there are no reports of incorporating nitride content into PEO-treated titanium alloys by modifying the electrolyte composition.

Cirrus aimed to design a single low power PEO process for light metal surface modification. Our mission was to improve the versatility and simplicity of PEO processes while staying ahead of the environmental and sustainability benchmarks. The current study addresses the need for surface treatment process that directly protect T1 titanium alloys with a thin ceramic oxide coating using low energy and benign electrolytic chemicals. The PEO surface treatment methodology described here produces functional ceramic coatings on T1 Ti alloys using voltages less than 120 V and environmentally friendly silicate-containing organo-alkaline baths. Our PEO technology produces nitrogenated coatings in electrolytes containing trace amounts of aminophenol^20^. The high-growth rate coatings (~ 40 μm/h) are composites of titanium oxides, carbides, nitrides, and silicate compounds. We were particularly interested in analysing the influences of carbide and nitride compounds on the functionality of the ceramic coatings. To the best of our knowledge, there have been no previous studies on combining silicates and nitrogen-based compounds in a single PEO electrolyte to produce functional titanium oxide-based composite coatings on T1 Ti alloys.

## Results and discussion

### Morphological distinction

**Figure 1:**
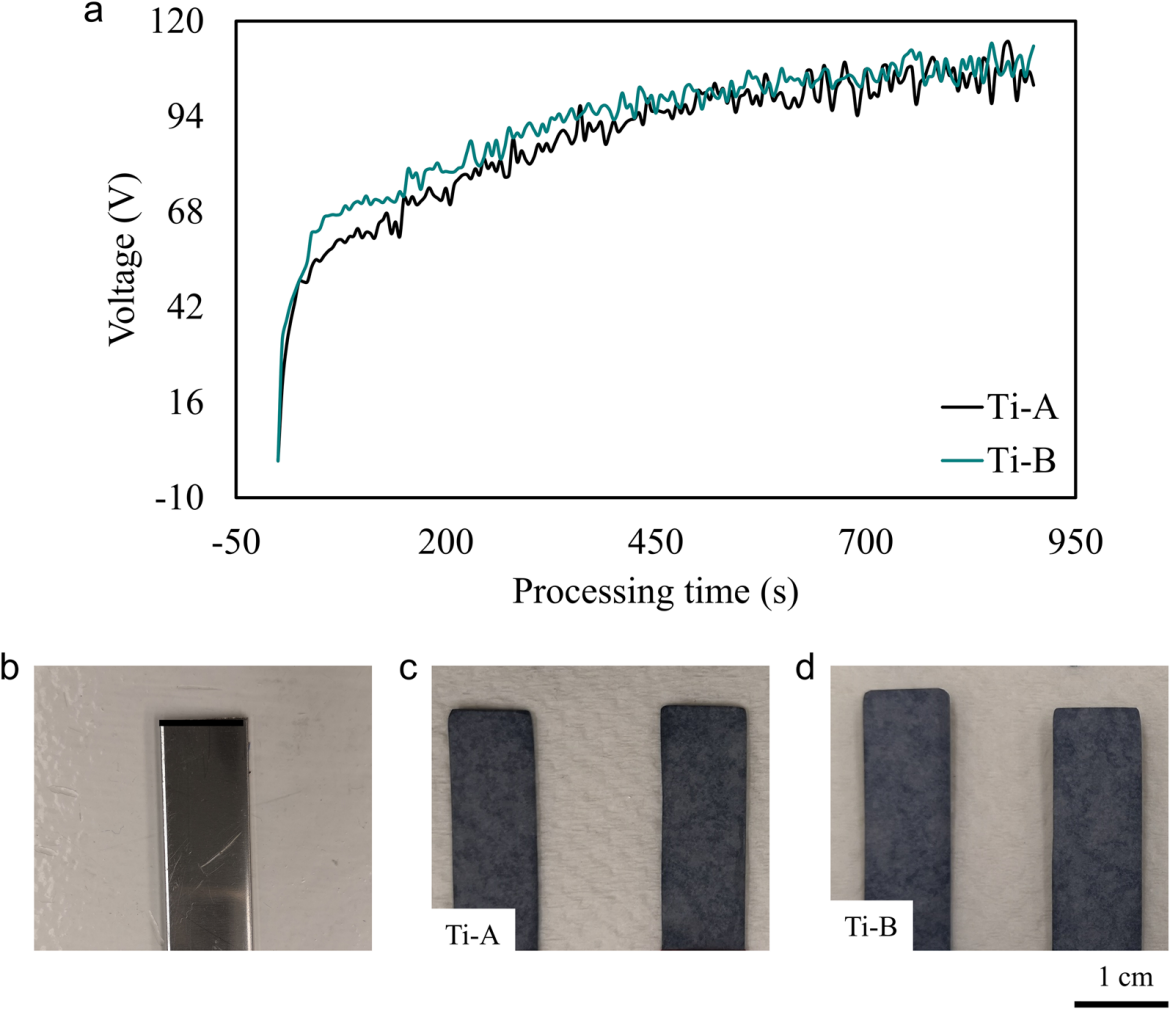
(**a**) Voltage vs time graph demonstrating the low energies required to produce oxide coatings on T1 Ti alloys using eco-friendly PEO electrolytic baths. (**b**) As-received T1 Ti alloy machined to 8 mm × 20 mm × 0.1 mm substrate dimensions for PEO treatment. (**c**) PEO-treated T1 alloys with silicates in the organo-alkaline bath. The ceramic coating, Ti-A, is continuous and bluish grey in appearance. (**d**) PEO treated T1 alloys with silicates and aminophenol in the organo-alkaline bath (Ti-B). The coatings, Ti-A and Ti-B, appear to be visually similar. Photo credit: F. Hou (**b**–**d**).

Figure 1a shows the variation of voltage during the PEO treatment of the T1 titanium alloys. The unique feature of this process is that the surface treatment requires less than 120 V. We treated two sets of T1 titanium alloys using the PEO technology reported in this paper. Figure 1b is an image of as-received T1 titanium alloy machined to the required dimensions. Samples labelled Ti-A (Fig. 1c) were prepared using silicate-containing organo-alkaline baths. PEO treatment caused the samples to develop a uniform bluish-grey ceramic coating on the surface. Samples prepared using silicate and aminophenol in the organo-alkaline bath chemistry are labelled Ti-B (Fig. 1d).

**Figure 2:**
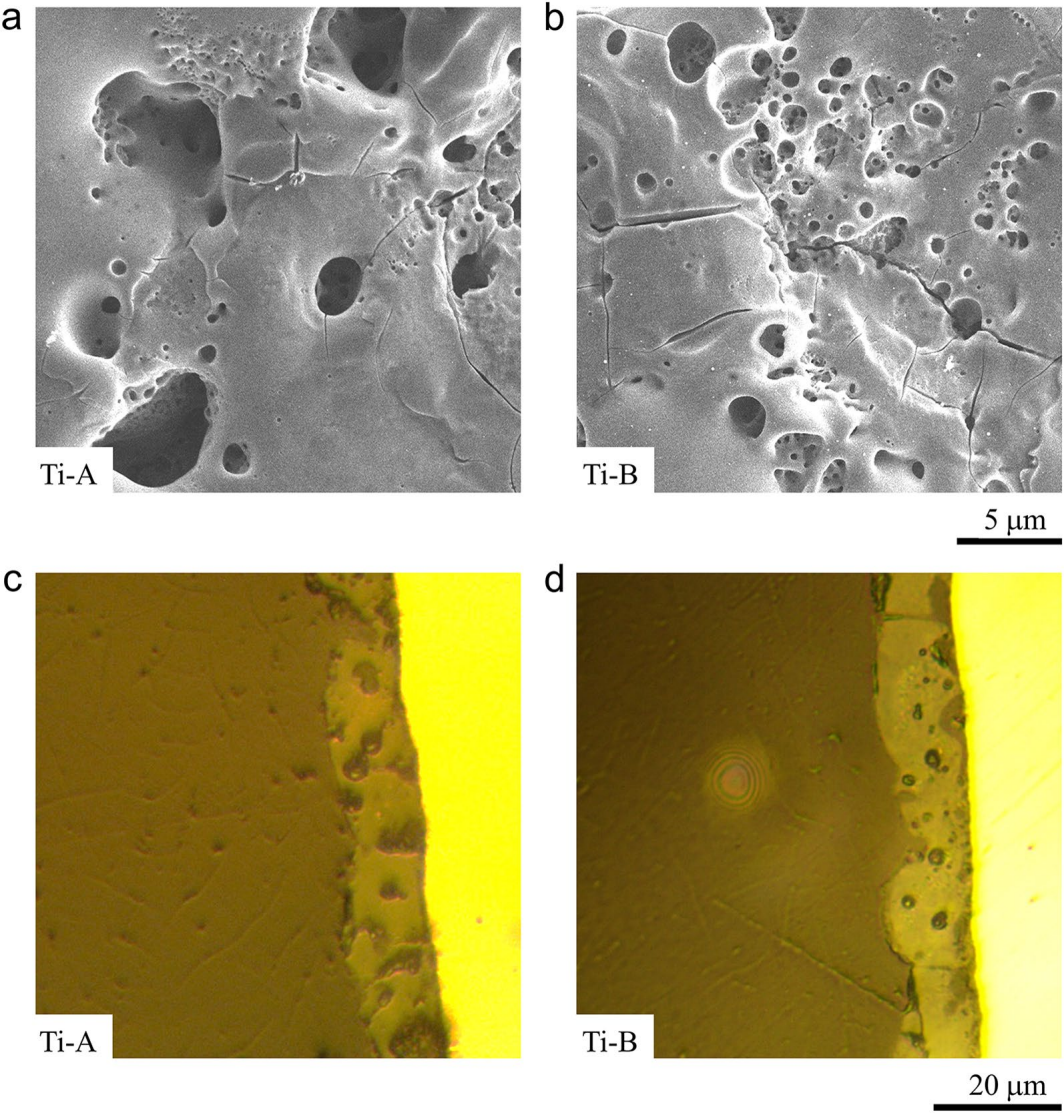
(**a**) Surface morphology of Ti-A: the figure shows the porous oxide layer on T1 alloy treated in PEO bath. The cracks and variation in the pore sizes are typical of a treated Ti surface. (**b**) Surface morphology of Ti-B: addition of aminophenol to the PEO bath modified the pore structure of the PEO-treated Ti coating. The pores are relatively uniform in size and appear to have a more uniform distribution on the surface. (**c**) Cross-sectional morphology of Ti-A showing the pore-distribution along the depth of the coating. (**d**) Cross-sectional morphology of Ti-B; the pore distribution is more uniform compared to Ti-A. Additionally, the image also shows the propagation of surface cracks through along the depth of the coating.

Macroscopically, Ti-A and Ti-B possess similar appearing bluish-grey ceramic coatings. Scanning electron microscopy (SEM) images of the Ti-A and Ti-B surfaces showed that the addition of aminophenol to the PEO bath induced microscale morphological changes to the surfaces. Ti-A surface (Fig. 2a) exhibited cracks and pores typical of a ceramic oxide coating^16,21^. The pore sizes ranged from < 1.00 to 3.00 μm. The image also shows that the Ti-A pore distribution is non-uniform as illustrated by cross-sectional image provided in Fig. 2c. The surface for Ti-B (Fig. 2b) has cracks and pores integral to oxidized layers but the pore-structure here appears denser. The formation of oxide films during PEO treatments is a facile yet complex process. The application of voltage between the Ti substrate and the electrolyte initially leads to formation of an insulating barrier layer on the surface. Once the applied voltage exceeds the breakdown potential of the barrier layer, conductive discharge channels form. Elevated temperatures in the discharge channels lead to the melting and oxidation of subsurface alloy elements. Molten oxides transported through the discharge channels instantaneously solidify producing the basalt-like morphology observed on Ti-A and Ti-B coatings. The coatings thicken as the process continues. Growth kinetics of PEO-treated surfaces suggest that porosity of the coatings increases linearly with thickness^22^. In this study, we observe that the addition of aminophenol to PEO electrolyte refines the porosity without influencing the growth-rate of Ti-B coating. The electrolyte modification enabled a greater control in pore dimensions on Ti-B surface due to the surfactant-like behaviour of aminophenol. Our hypothesis is that reduction in the gas bubble dimensions is due to variations in arc-discharge intensity, temperatures and pressures in the discharge channels caused by aminophenolic modification of the PEO electrolyte. The changes in electrolyte properties also appear to increase the uniformity and density of discharge channels on the surface. The result—a high porosity coating with controlled morphology observed in Ti-B (Fig. 2b)^23^. The reduction in pore dimensions result in less molten oxide transport per discharge channel. The higher porosity on Ti-B coating, manifesting as more prominent surface cracks, may influence the corrosion properties. Figure 2d shows propagation of surface cracks on Ti-B through the thickness of the coating. The modified microstructure of Ti-B coatings, due to addition of nitrogen-containing compounds in the PEO bath, could potentially enhance the mechanical performance of the coatings.

### Compositional characterization

**Figure 3:**
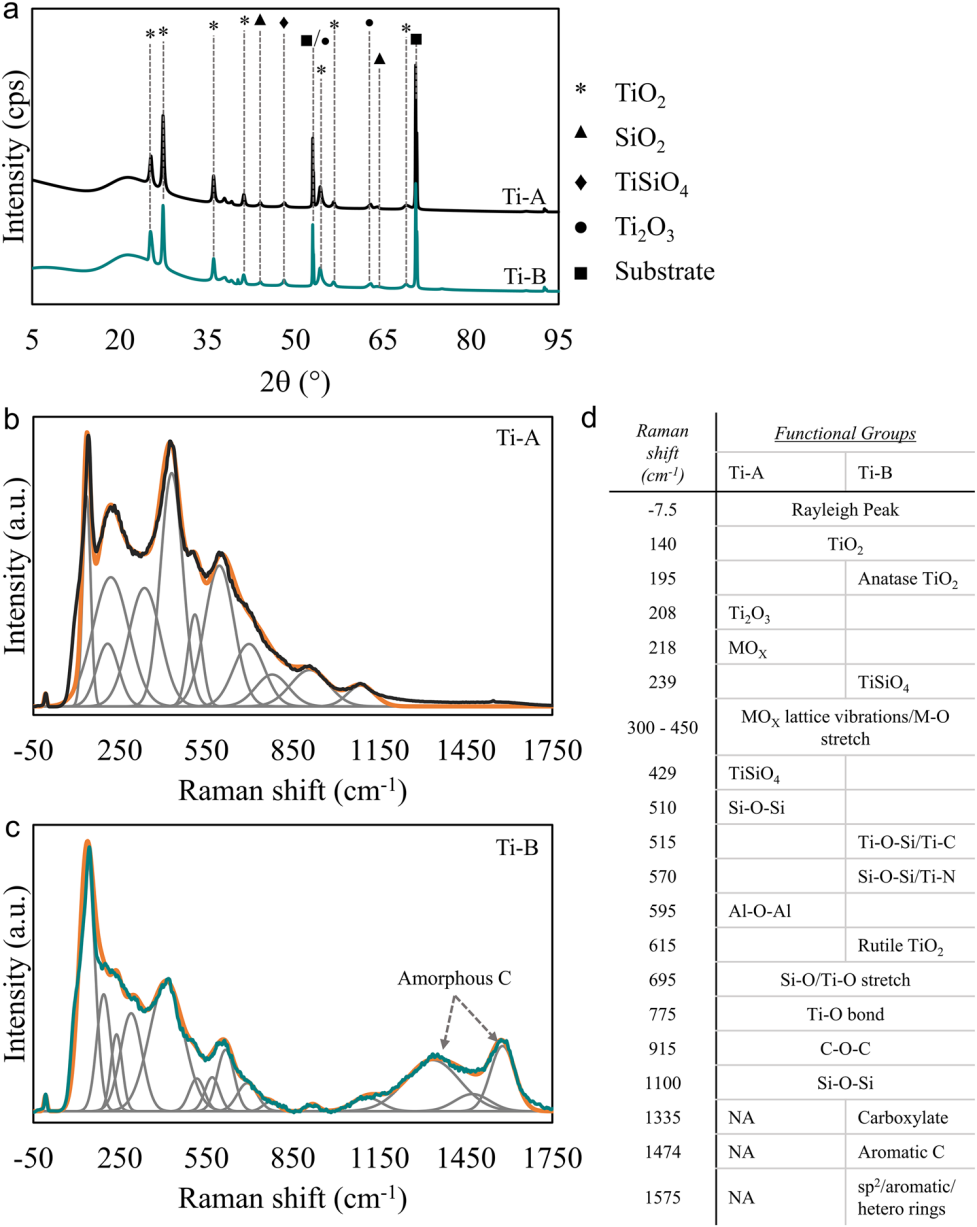
(**a**) XRD patterns of Ti-A and Ti-B. The patterns show that the addition of aminophenol to the bath chemistry has no influence on the crystallographic signature of the formed oxide layer. (**b**) Raman spectrum of Ti-A with peak deconvolution. (**c**) Raman spectrum of Ti-B with peak deconvolution. The amorphous carbon signature detected in the oxidized layer is due to the addition of aminophenol to the PEO bath. (**d**) List of functional groups associated with the Raman bands observed in deconvoluted SERS spectra.

We characterised the crystallographic and compositional properties of Ti-A and Ti-B using X-ray diffraction (XRD) and surface enhanced Raman spectroscopy (SERS). We compared the XRD patterns of Ti-A and Ti-B coatings with that of an untreated Ti alloy to isolate the signals from substrate. XRD patterns in Fig. 3a show that surfaces oxidised in PEO baths containing aminophenol exhibit similar crystallographic signatures to those treated in baths without aminophenol. Most of the peaks correspond to anatase and rutile phase TiO_2_. The peaks for TiO_2_ and Ti_2_O_3_ appear to coincide at ~ 53°.

XRD patterns for Ti-A and Ti-B are similar, thus indicating that aminophenol modification of the electrolyte has little influence on the crystallographic properties of the coatings. A future study is focused on the possible enhancement of crystal plane orientation due to the influence of substrate crystallography, electrolyte composition and coating growth-rate. Coating growth-rates in PEO processes are high for silicate-containing electrolytes^24^. However, the current results do not provide us with sufficient data to analyse the crystallographic orientation.

Figure 3b,c illustrate the Raman spectra of Ti-A and Ti-B samples treated in PEO baths without and with aminophenol, respectively. Figure 3d provides a list of functional groups associated with the deconvoluted peaks. The spectral deconvolutions for Ti-A and Ti-B spectra show that the coatings are composites of aluminium, silicon, and titanium oxides, titanium silicate and organic compounds. Additionally, we detect amorphous carbon in the Ti-B coating. Ti-A and Ti-B exhibit a sharp peak for anatase phase TiO_2_ at ~ 140 cm^−1^. This compliments the observation of oriented anatase crystal planes from XRD patterns. The detection of TiSiO_4_ concurs with the XRD results for both Ti-A and Ti-B coatings^25^. The spectrum for Ti-B (Fig. 3c) shows that the oxide coating contained amorphous carbon and other organic compounds. The appearance of the amorphous carbon peaks in the spectrum suggested the potential carbonization of the oxide layer during the PEO process. The peaks at ~ 515 cm^−1^ and ~ 570 cm^−1^ suggest the presence of TiC^26^ and TiN^27^ in the Ti-B coatings. The absence of peaks corresponding to TiC in the XRD pattern for Ti-B is due to insufficient intensity from carbide particles for reliable detection. Another possibility is that the TiC particles are not oriented to the direction of the incident X-rays.

**Figure 4:**
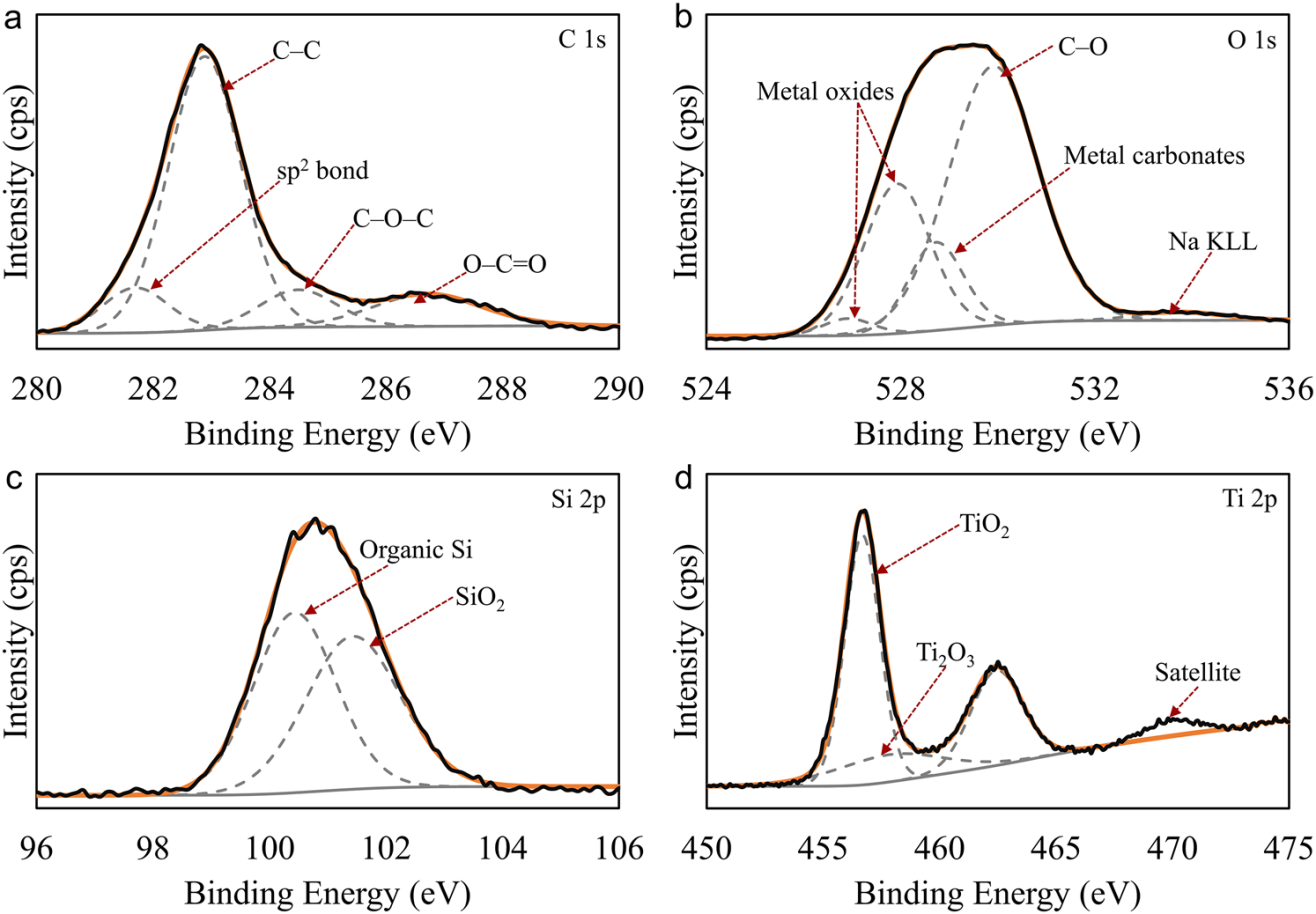
XPS core-loss spectra for Ti-A. (**a**) XPS spectrum showing the C 1s peak located at ~ 283 eV. The signature primarily corresponds to adventitious carbon. We observe a low intensity peak corresponding to polymeric C=O at ~ 287 eV. (**b**) Deconvoluted XPS peaks for oxides in the PEO-treated coating. The peaks at ~ 529 eV are signals from adventitious C=O and C–O and from any metal carbonates in the coating. (**c**) Deconvoluted Si 2p peaks showing signatures for organic Si (~ 100.4 eV) and SiO_2_. (**d**) We observe XPS peaks for TiO_2_ and Ti_2_O_3_ in the core-loss spectra collected for Ti 2p. Also observed in the figure is the satellite feature at ~ 471 eV typical of titanium oxides.

We used X-ray photoelectron spectroscopy (XPS) for advanced analysis of Ti-A and Ti-B samples. We collected core-loss spectra for C, N (Ti-B only), O, Si and Ti. Figure 4 provides Ti-A core-level spectra. Peak analysis for the samples referenced the C 1 s peak at 283 eV (Fig. 4a). The peak deconvolutions exhibited the presence of C–C bonds at ~ 283 eV and C–O–C bonds at ~ 284 eV both of which correspond to adventitious carbon content accumulated on the coating surface. We observed a low-intensity broad peak corresponding to carboxylate bonds at ~ 287 eV. Ti-A samples also contained minute amounts of sp^2^ bonded carbon in the oxidized coating. We hypothesize that the citrates in PEO bath have the potential to carbonize the ceramic coating to a small extent. Deconvoluted O 1s peaks (Fig. 4b) showed the presence of metal oxides at ~ 528 eV, metal carbonates at ~ 529 eV, and C–O bonds ~ 530 eV. The peak at 530 eV corresponds to adventitious content accumulated on the coating surface. The low intensity peak at ~ 534 eV corresponds to Na KLL. Si 2p spectrum (Fig. 4c) deconvolution resulted in two peaks. The peak at ~ 101 eV corresponds to SiO_2_ and the one at ~ 100 eV indicates the presence of organic silicon content in the coating. Ti 2p spectrum (Fig. 4d) exhibited a shape normally representative of TiO_2_ with a small amount of Ti_2_O_3_. Surface compositional analysis indicated that Ti-A samples are composite ceramic coatings prepared in a PEO bath capable of introducing mechanically strengthening Si–O-containing compounds. We added nitrogen-containing compounds to the bath chemistry with an aim to further enhance the mechanical and functional properties of the oxide coatings.

**Figure 5:**
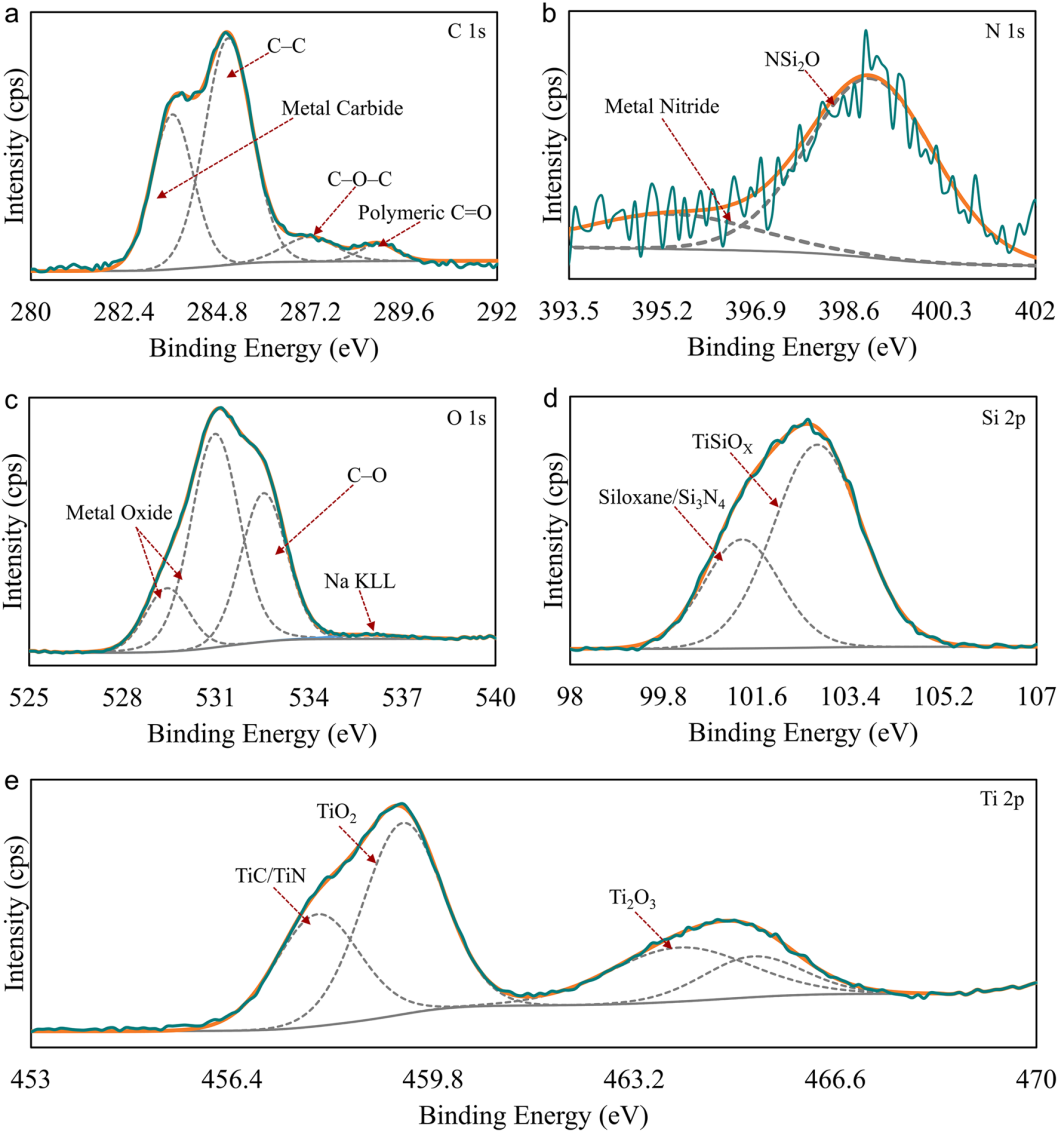
XPS spectra for Ti-B. (**a**) The C 1s peak for the coatings oxidized with aminophenol in the PEO bath is at ~ 284 eV. The peak at ~ 282 eV indicates the presence of Ti-C in the coating. (**b**) Core-loss peak for N 1s shows the presence of metal nitrides in the coating, potentially synthesized under the influence of arc energy in generated by nitrogen-containing PEO bath. Additionally, we also observe a low intensity peak for NSi_2_O at ~ 399 eV. (**c**) XPS peaks for O1s correspond to the presence of metal oxides and adventitious material on the coating surface. (**d**) XPS spectra for Si2p indicate that the ceramic layer contains TiSiO_X_ and potentially Si_3_N_4_. (**e**) Ti2p core-loss peaks exhibit a complex shape. The coating is primarily composed of titanium oxides. The minor satellite feature at ~ 459 eV is due to the presence of Ti–N bonds in the coating. The peak at ~ 455 eV corresponds to the presence of Ti–C bonds.

Figure 5 provides XPS core-level peaks for Ti-B samples. The shapes of peaks for C, O, Si and Ti are noticeably different between Ti-A and Ti-B. Figure 5b,e indicated the nitrogenation of the coating composition. We hypothesize that the incorporation of nitrides in Ti-B coatings is due to the influence of arc energy generated at the alloy surface in the nitrogen containing PEO bath. We analysed XPS core-level spectra for Ti-B with reference to the C 1s (C–C) peak at ~ 284 eV (Fig. 5a). Deconvolution of C 1s spectrum resulted in four distinct peaks; the first peak at ~ 282 eV suggests the presence of metal carbides and the one at ~ 287 eV indicates the presence of polymeric C=O bonds in the Ti-B coatings. The other two peaks, C–C at ~ 284 eV and C–O–C at ~ 285 eV, correspond to the presence of adventitious carbon content on the coating surface. XPS spectrum for N 1s in Ti-B coatings exhibited two deconvoluted peaks (Fig. 5b); the peak at ~ 396 eV suggests the presence of a small amount of metal nitride in the composition. The peak at ~ 399 eV indicates that most of the nitrogen signature is from silicon oxynitride content in Ti-B coatings. O 1s spectrum for Ti-B has a complex shape and the peak deconvolution resulted in four peaks (Fig. 5c). The peaks at ~ 527 eV and ~ 529 eV indicate the presence of metal-oxides in Ti-B coatings. The peak at ~ 531 eV corresponds to the C–O bonds in the coating typical of adventitious contamination. Finally, we observe the Na KLL peak at ~ 535 eV. The Si 2p core-level spectrum collected from Ti-B samples had a slightly asymmetrical shape (Fig. 5d). Peak deconvolution showed that the Si content in the coatings is primarily in the form of TiSiO_X_ (~ 102 eV). The deconvolution also suggested the presence of of Si_3_N_4_ (~ 100 eV). Figure 5e shows the XPS spectrum for Ti 2p in the Ti-B coatings; the spectrum has a complex shape and the deconvolution resulted in four distinct peaks. The peaks at ~ 457 eV and ~ 463 eV correspond to the Ti 2p^3/2^ and Ti 2p^1/2^ in TiO_2_. The peak at ~ 461 eV corresponds to Ti_2_O_3_. The interesting feature in this spectrum is the peak at ~ 455 eV suggesting the presence of TiN^27^ and TiC^28^ in the coating. These results support the XRD, and SERS data presented for Ti-B in Fig. 3a,c.

The material transfer through the discharge channels generated during PEO treatments is bi-directional. We state in earlier sections that the molten oxides transport occurs from the subsurface to the surface. The localized elevated temperatures decompose the electrolyte constituents leading to the incorporation of polycondensed silica ions into the discharge channels. We assume this phenomenon is also responsible for incorporation of organic compounds (citrates) into Ti-A and Ti-B coatings. Decomposition of aminophenol, in the discharge channels, explains the incorporation of nitride and carbide content into Ti-B. Most research studies rely on this mode of particle incorporation to enhance the functional, mechanical, and electrochemical properties of PEO-treated surfaces^29^.

### Mechanical behaviour

We evaluated the mechanical properties of Ti-A and Ti-B coatings using nanoindentation. The results showed that the PEO process reported in this paper improved the hardness by approx. 1.5 times. Bare Ti-alloys possess hardness of 4.43 GPa. The coatings oxidized with and without aminophenol in the bath chemistry exhibited hardness values of 7.12 GPa and 6.63 GPa, respectively. This is a clear demonstration of the well-known enhancement in mechanical behaviour of T1 substrates via PEO-assisted surface modification^30^. The improved hardness of the treated alloys is due to the formation of a robust oxide layer reinforced by silicate particle incorporation. It is possible that the cracks observed on Ti-A and Ti-B are a result of silicate-induced brittleness^24,31^. TiC and TiN content in Ti-B coatings are responsible for their high hardness value^32^.

### Functional properties

**Figure 6:**
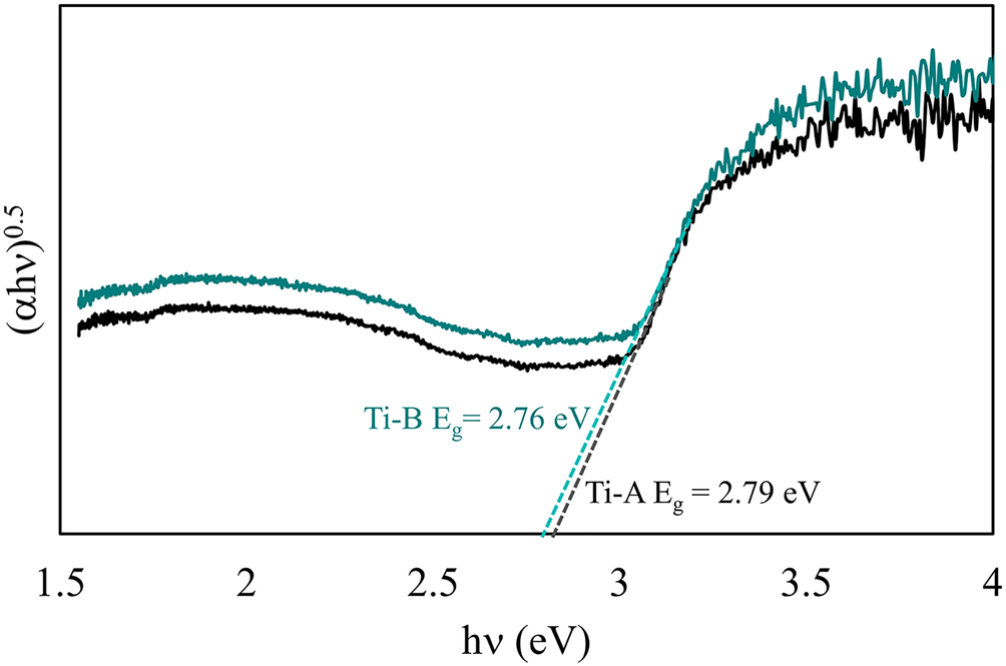
Diffuse reflectance spectra of Ti-A and Ti-B coatings. Spectra show that the coatings exhibit bandgaps in the visible blue light region (~ 446 nm). There is no variation in the bandgap for samples oxidized in PEO baths with and without nitrogen-containing compounds.

Figure 6 shows the diffuse reflectance spectra for Ti-A and Ti-B coatings prepared by surface treating T1 alloys in PEO baths with and without nitrogen-containing compounds. We used Kubelka–Munk theory to transform the reflectance spectra for generating Tauc plots. We assumed Ti-A and Ti-B coatings to possess direct bandgaps. The bandgap estimations of 2.79 eV for Ti-A and 2.76 eV for Ti-B are remarkably lower than commercially available TiO_2_ (3.0 to 3.2 eV). The results show that the coatings are potentially photocatalytic in the low-energy visible blue light (~ 446 nm) region. The smaller bandgap of Ti-A and Ti-B coatings is due to their silicate and organic content. Vasilyeva et al. report that silica/titania composites exhibit catalytic conversion properties with 97% efficiency; while nitrogen-doping^33^ and carbonization^34^ of titanium oxide-based coatings are popular routes of enhancing their functional properties for various semi-conductor applications. The current bandgap reduction is limited, yet the process shows promise for developing photocatalytic materials. We are currently studying the coatings’ photocatalytic efficiency.

### Corrosion behaviour

**Figure 7:**
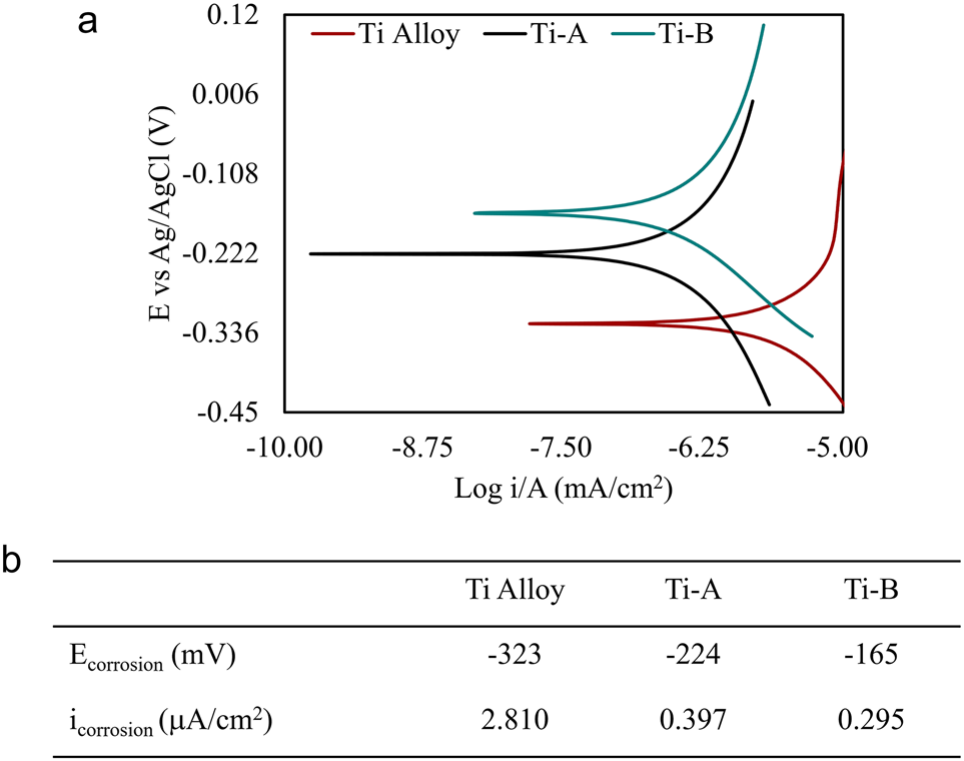
(**a**) Tafel plots for Ti-A and Ti-B coatings. The curve for a bare T1 titanium alloy was also measured as a baseline. (**b**) The corrosion potentials and current densities extracted from the curves show that Ti-B is less susceptible to corrosion than Ti-A and the untreated Ti alloy.

We extracted the corrosion potentials of the Titanium substrate, and the Ti-A and Ti-B coatings from the Tafel plots (Fig. 7). Figure 7b provides the extracted potentiodynamic test results. The Tafel results show that the untreated Ti alloy displays a less noble corrosion resistance as compared to Ti-A and Ti-B. Ti-A coating yields a less noble corrosion behaviour than Ti-B. The Tafel plots indicate the presence of nitrides and carbides in Ti-B have a positive influence on corrosion behaviour. SEM images of Ti-A and Ti-B (Fig. 2) reveal that the coatings are porous with surface cracks. These can propagate through the thickness of Ti-B coating and may negatively impact its corrosion resistance. Dissimilar materials combined with porosity in both Ti-A and Ti-B coatings may have a detrimental influence due to galvanic corrosion. Further work is required to optimize the process parameters to reduce the porosity of the surface coating to further improve their corrosion resistance.

It is important to acknowledge that while the i_corrosion_ values are reported as current densities, this is done under the assumption that the entire exposed surface area (1 cm^2^) in the electrochemical cell is active. The increased surface area due to the porosity was not measured, therefore, real-world corrosion current densities are likely lower than the values reported in Fig. 7b.

**Figure 8:**
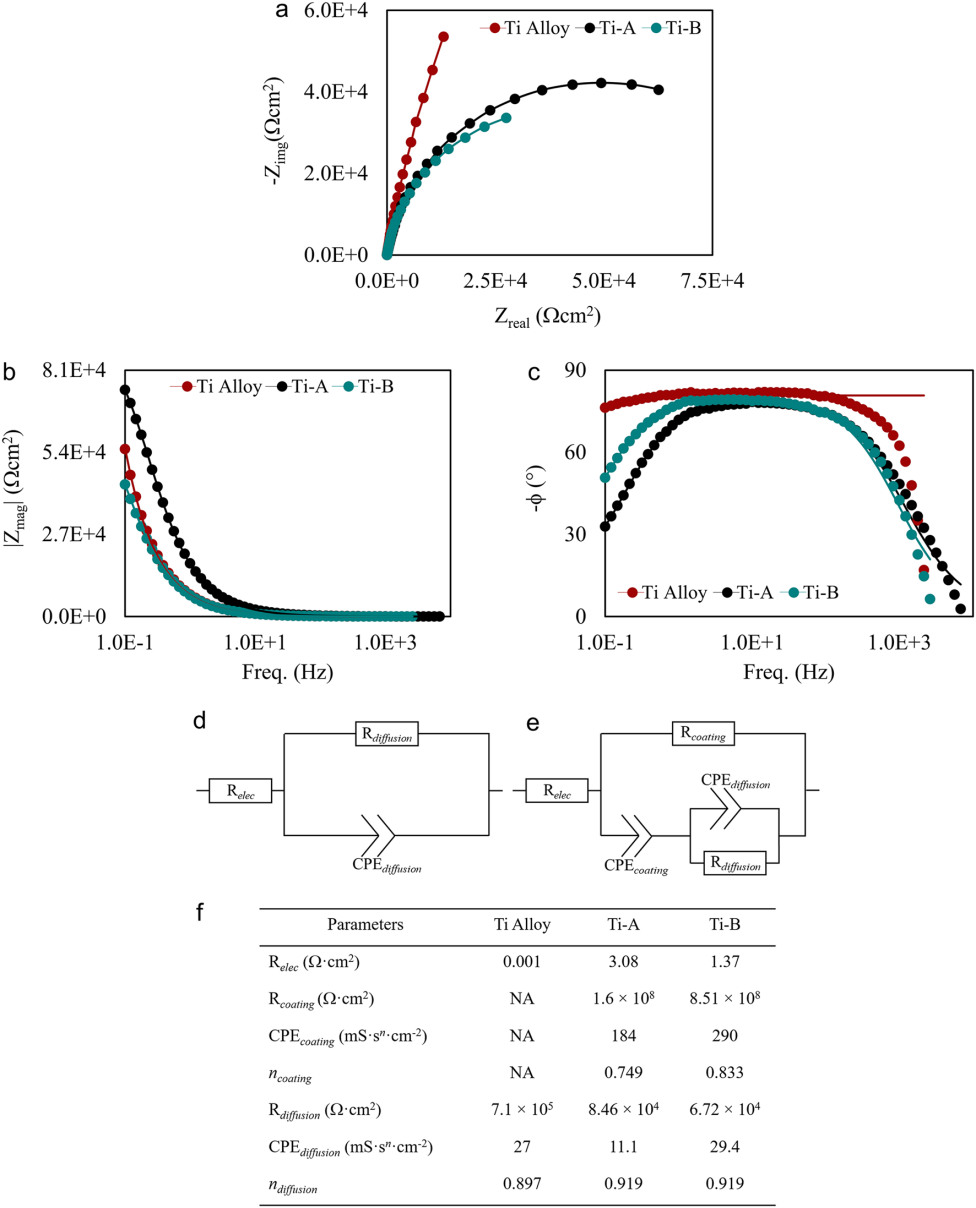
Electrochemical impedance spectroscopy of Ti-A and Ti-B coatings compared to the electrochemical behavior of an untreated Ti alloy. (**a**) Nyquist plots; (**b,c**) Bode plots; (**d**) EEC model for untreated Ti alloy; (**e**) EEC models for Ti-A and Ti-B; (**f**) list of fitting parameters of EIS EECs obtained for untreated Ti alloy, Ti-A and Ti-B.

Figure 8 demonstrates the impedance behaviour of PEO-treated Ti alloys compared that of untreated Ti substrates. We modelled the electrochemical impedance spectroscopy (EIS) response of untreated and PEO-treated substrates using the equivalent electrical circuits (EEC) illustrated by Fig. 8d,e. R_*coating*_, R_*diffusion*_, and R_*elec*_ represent the coating, diffusion layer and electrolyte resistances respectively. CPE_*coating*_ and CPE_*diffusion*_ represent the constant phases elements (CPE) for coating and diffusion layers. The modelling of EIS results as an EEC is common for coatings on metal substrates. The porosity is represented by the parallel CPE_*diffusion*_ and R_*diffusion*_ elements (Fig. 8e). Comparing Ti-A and Ti-B, the magnitude of impedances from each of the fitting elements (Fig. 8f) is seen to be larger for Ti-B, suggesting that addition of aminophenols has a positive influence on the corrosion resistance of Ti-B coatings.

Pores, stress-induced cracks, and highly porous diffusion layers are typical of a PEO-treated metal substrate. One of our aims for using an aminophenol as PEO electrolyte modifier is to limit the formation of coating defects through the treatment process. We observed that nitridation and carburization of PEO treated coatings densifies the coating structure and mitigates the propagation of stress induced cracks (Fig. 2b,c). This phenomenon is evident from the EEC modelling parameters. The resistance of the Ti-B coating is approximately 5 times higher than Ti-A, but the diffusion layer resistance of Ti-B is lower than Ti-A. Hoche et al. proposed a model that explains the observed behaviour of our PEO-treated coatings^35^. The NaCl solution first penetrates the process-induced defects. The larger pore dimensions and propagating surface cracks on Ti-A are responsible for the poorer corrosion resistance of Ti-A than Ti-B. We also observe that the addition of aminophenols to PEO electrolytes increases the distribution of arc discharge channels on the substrate surface leading to a poorer corrosion resistance observed in Ti-B diffusion layer.

## Summary

The objective of this study was to demonstrate the application of eco-friendly PEO bath chemistries in surface treatment of T1 Ti alloys. The surface treatment process described here produced uniform and adherent coatings on the substrate surface with interesting characteristics. The process also offered a single-step technique to incorporate nitride and carbide content into the surface oxides. Our bandgap analysis tests showed that the surface oxides produced during the PEO treatment process may have visible light photocatalytic applications. The oxide layers formed during PEO provided some unique corrosion-resistant properties to the T1 titanium substrates.

The morphology and composition of surface oxides using PEO baths were attributed to the non-equilibrium behaviour of reactive species generated under the influence of localized micro-arc discharge points at the T1 titanium alloy surfaces. PEO processes generate intense localized thermal energy fluxes that have a bi-modal influence on T1 Ti substrates. The direct oxidation of the substrate forms the porous structure of the oxide coating. The temperatures (~ 3000 °C)^36^ achieved during the PEO treatment process were sufficient to melt the substrate surface and create the basalt-like appearance of the oxide layers. The uniform pore distribution in the surface oxide layer was important for the coating to protect the underlying substrate from corrosion. We found that surface treatment with nitrogen compound-containing PEO bath has improved the pore density of the resultant oxide layer while preserving the basalt-like morphology of the coating. However, the hardening of the Ti-B coatings produced using the aminophenol-enhanced PEO bath has also caused stress-induced cracks to appear on the surface.

The novelty of the Ti-B coatings lies in the fact that adding nitrogen-containing compounds to the bath chemistry rather than C^4−^- or N^3−^-based nanoparticles has improved the uniformity in pore distribution. Our hypothesis is that the presence of nitrogen-based chemicals in the bath chemistry caused a phenomenon similar to the nitrogenation treatment reported by Shen et al.^37^. The results from compositional analyses presented in this paper show that nitrogenation of the PEO-treated surfaces can be achieved using eco-friendly chemicals and at low oxidation voltages. Carburization of coatings was also achieved without the post-processing the PEO-treated surfaces^19^. To the best of our knowledge, there are no other reports on the preparation of TiC containing PEO coatings using organic electrolytic baths.

Referring to the SEM images of Ti-A (Fig. 2a) and Ti-B (Fig. 2b) it appears that Ti-B has cracks which reduced the corrosion protection of the PEO coating in comparison to Ti-A. The presence of carbides and (oxy-)nitrides in the coating, as shown in the XPS measurement data (Fig. 4), would indicate more brittle mechanical properties. The mechanical testing showed the Ti-B coating to be approximately 7.5% harder than Ti-A. The surface carbides and (oxy-)nitrides will introduce brittle character to the Ti-B coating which would explain the many cracks visible in the Ti-B coating.

Ti-A and Ti-B coatings exhibit bandgaps of < 2.8 eV suggesting that the materials have visible light (λ = ~ 440 nm) photoactivity. This remarkable reduction of TiO_2_ bandgap from ~ 3.1 eV is due to the presence of silicate and carbon-containing compounds in Ti-A and silicate, carbon, and nitrogen-containing compounds in TiB. Similar functionalization of PEO-treated titanium alloys, reported by few other research groups, was only obtained by incorporating nitride or carbon-containing compounds prior to or post PEO-treatment. Lin et al. produced N-doped TiO_2_ visible light photocatalysts using a TiN seed layer on Ti foils subjected to PEO surface treatment^38^. Tao et al. functionalized PEO-treated Ti alloys by thermal polycondensation of graphitic C_3_N_4_ onto the oxidized substrates^39^.

Cirrus PEO technology has an overall positive influence on the electrochemical behaviour of treated Ti substrates. Stress-induced cracks in Ti-A and Ti-B may be detrimental to the corrosion resistance of the coatings. The potentiodynamic behaviour of Ti-A coatings was in accordance with similar materials reported in the literature^2^. The much lower corrosion of Ti-B compared to Ti-A is attributed to the reduction in pore dimensions and densification of the coating due to nitridation and carburization.

## Conclusion

The results presented in this paper suggest that light metals can be reliably protected using low energy PEO processes and eco-friendly electrolytes. We demonstrated a facile method to incorporate carbon and nitrogen-compounds into titanium oxide coatings to achieve visible light functionalization. The corrosion behaviour of the coatings was unexpected and requires further research. We are currently working on mitigating stress-induced cracking of the nitride and carbide-coatings during PEO processing. We are currently researching the applicability of the PEO technology (patent pending) reported in this paper for a wide range metal alloys such as Mg, Al, and mild steel.

## Methods

### Substrate preparation and pre-treatment

We used T1 Ti alloys measuring 20 mm × 8 mm × 0.1 mm in dimensions the study reported here. Prior to treatment by PEO the sample surfaces were prepared by mechanically roughening the substrate using an emery paper followed by cleaning in an alkaline bath, maintained at 80 °C, for 15 min. The bath comprised 20 g/L NaCO_3_, 20 g/L Na_2_PO_4_, 20 g/L Na_2_SiO_3_, and 3 g/L commercially available OP-10 surfactant. Finally, we rinsed the substrates with DI water before treating them in the PEO baths. Mechanical roughening improved the adhesion between the ceramic coating and the substrate. We ensured that the cleaning step prevented any build-up of the native oxide layer on the substrate.

### PEO process

We treated two sets of Ti-alloys, Ti-A and Ti-B, in a PEO bath consisting of 70 g/L NaOH, 60 g/L Na_2_SiO_3_, 10 g/L Na_3_C_6_H_5_O_7_, 6 mL/L H_2_O_2_, and 0.05 milli mol/L sodium dodecyl sulphate. The bath for Sample set ‘B’ contained 4.9 mL/L aminophenol in the bath. We maintained both electrolyte baths at ~ 25 °C. We treated Ti-A and Ti-B samples using a stainless-steel counter electrode for 15 min. A variable DC power supply supplied a constant current of 4 A/dm^2^ resulting in an average processing voltage of < 120 V.

### SEM–EDS

We used an FEI XL30 equipped with a 30 kV field emission gun to study the surface morphologies and composition of Ti-A and Ti-B coatings. We sputtered the samples with Pt using a Quorum Tech Q150T turbomolecular pumped coater to improve their electron conductivity for SEM imaging.

### XRD

We collected phase and composition data from Ti-A and Ti-B using a Rigaku XtaLAB Synergy-s single crystal X-ray diffractometer equipped with a Cu K_α_ source (λ = 1.54184 Å, 2θ = 20° to 80°, 0.02° step size). We used the Materials Explorer application on Materials Project open database^40^ to index the XRD patterns.

### SERS

We collected SERS spectra for Ti-A and Ti-B coatings using a WITec alpha300 Raman-AFM spectrometer equipped with a λ = 532 nm Ar-ion laser (− 61 °C). The spectral data was collected using an integration time of 10 s each for five accumulations.

### XPS

We used a Kratos AXIS DLD X-ray photoelectron spectrometer with a hemispherical electron energy analyser for analysing Ti-A and Ti-B coatings on Ti alloys. We generated the spectra using monochromatic Al K_a_ X-rays (1486 eV) operated at 150 W. The analysis chamber was at 1 × 10^−9^ Torr for data collection. We used the survey scans from − 5 to 1350 eV (160 eV pass energy) to determine the material composition. We collected core level data for C, O, Si and Ti for all the samples, and for N from Ti-B samples. We collected the core level data with a pass energy of 20 eV.

We used Casa XPS 2.3.14 to resolve the XPS data by aligning the C 1s peak at 285 eV. We used Thermo Scientific XPS database to analyse the deconvoluted peaks^41^.

### Nanohardness behaviour

We used a Hysitron TI 950 tribometer equipped with a Berkovich tip to analyse the mechanical behaviour of the ceramic oxide coatings. We applied a maximum load of 1000 mN for 2 s with 5-s preloading and unloading times. We assessed the nanohardness of Ti-A and Ti-B coating cross-sections eliminate any influence of the substrates on the results. We evaluated the nanohardness of untreated control substrates to evaluate the mechanical enhancement provided by the ceramic coating.

### Bandgap analysis

We collected diffuse reflectance spectra for Ti-A and T-B coatings using a Shimadzu 2250 UV–Vis spectrophotometer. The photonic energies ranged from 1.5 to 6.2 eV (λ = 200 to 800 nm) with a medium scan speed and 0.016 eV (0.5 nm) resolution. We irradiated the flat samples with a 5° photonic incidence angle via the specular reflectance attachment.

### Corrosion resistance

We used a CH Instruments three-cell electrochemical workstation equipped with a Metek designed K0235 flat cell kit to evaluate the electrochemical performance of the oxidized surfaces. Prior to testing, we immersed the samples in 3.5 wt% NaCl solution for 72 h. The tests employed an Ag/AgCl reference electrode and a Pt counter electrode. We collected Tafel data using freshly prepared 3.5 wt% NaCl solution. We determined the open circuit potential (OCP) of Ti-A and Ti-B coatings by scanning them between ± 0.3 V for 5 min. We also performed Electrochemical Impedance Spectroscopy (EIS) applying an AC Voltage of 10 mV amplitude to the OCP, in the range of frequencies tested. An open-source Python package, impedance.py^42^, was used to analyse the resultant EIS spectra and develop electrochemical equivalent circuits.
